# Surgical Management of Choanal Atresia: Two Classic Cases and Review of the Literature

**DOI:** 10.7759/cureus.24259

**Published:** 2022-04-18

**Authors:** Nicholas A Rossi, Mia Benavidez, Harold S Pine, Shiva Daram, Wasyl Szeremeta

**Affiliations:** 1 Otolaryngology, University of Texas Medical Branch, Galveston, USA; 2 Surgery, University of Texas Medical Branch, Galveston, USA; 3 Pediatric Otolaryngology, Children's Ear, Nose and Throat (ENT) of Houston, Webster, USA

**Keywords:** surgeons, surgery, otolaryngologists, standard of care, endoscopy, charge syndrome, pediatric otolaryngology, choanal atresia

## Abstract

Choanal atresia is a rare congenital airway malformation that presents a unique surgical challenge for pediatric otolaryngologists. Here we report two classic cases of choanal atresia and examine the surgical approaches to this entity. The first case was a four-day-old female with a history of CHARGE syndrome and bilateral mixed membranous and bony choanal atresia confirmed by a CT scan. After undergoing transnasal endoscopic repair, choanal stents were placed for four weeks, and the patient was seen three months postoperatively and found to be doing well with no respiratory concerns. The second case involved a healthy three-year-old female presenting with unilateral combined membranous and bony atresia. Following successful endoscopic repair, she was seen at a three-month follow-up with no signs of restenosis. Additionally, a literature review was performed to evaluate updates since the 2012 Cochrane Review on surgical treatment of congenital choanal atresia.

## Introduction

Although choanal atresia has been described in the literature for over 250 years [1], there remains a lack of consensus between pediatric otolaryngologists regarding optimal surgical management. The most well-described options include the endoscopic transnasal, transpalatal, and transseptal approaches [1,2]. Other authors have explored the potential role of therapeutic adjuncts to surgery, such as drug-eluting stents, non-drug-eluting stents, or topical mitomycin C [3-10]. A 2012 Cochrane Review by Cedin AC et al. explored the evidence related to surgery for this congenital anomaly and found a preference for endoscopic transnasal repair [2]. However, a significant body of literature has emerged in the last decade with a growing emphasis on endoscopic surgery. This study aims to objectively examine the literature for updates on surgery for congenital choanal atresia since the Cochrane Review. In addition, we present two classic cases of choanal atresia, one unilateral and one bilateral, to highlight the contrasting clinical presentations of this entity treated with similar endonasal approaches.

Materials and methods

This study was a case series and review of the literature utilizing PubMed, Google Scholar, and Scopus databases. These were individually queried for articles with keywords “choanal atresia” and “surgery,” with a publication date between 2012 and 2021. Articles were examined and sorted by author, publication date, and summary of findings relevant to choanal atresia surgery.

A version of this project was previously presented at the 15th Congress of the European Society of Pediatric Otorhinolaryngology (Virtual, November 6-9, 2021).

## Case presentation

Case 1

**Figure 1:**
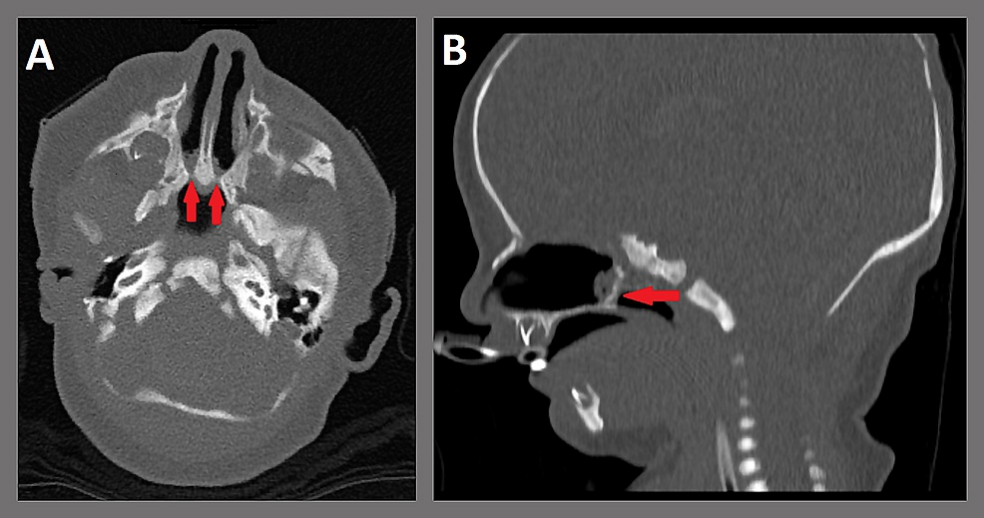
CT landmark sinus protocol without contrast (A: axial plane; B: sagittal plane). Note: Bilateral mixed membranous and bony atresia with a choanal size of less than 1 mm in diameter (red arrows).

A four-day-old female with a history of bladder exstrophy, ambiguous genitalia, and bilateral chorioretinal coloboma was seen in consultation in the neonatal ICU for inability to pass the nasogastric tube through the bilateral nares. The patient was intubated at the time of birth due to acute hypoxemic respiratory failure without response to conservative resuscitative measures. Physical examination revealed bilateral cup ear deformity. After suctioning bilateral nasal secretions, bedside flexible nasoendoscopy revealed complete bilateral choanal atresia. CT landmark sinus protocol without contrast revealed bilateral mixed membranous and bony atresia with a maximum choanal bony diameter of less than 1 mm (Figure 1). Thick mucinous secretions were admixed throughout the bilateral nasal cavities. Additionally, there was hypopneumatization of the ethmoid sinuses and a complete lack of pneumatization of the bilateral maxillary, sphenoid, and frontal sinuses. 

**Figure 2:**
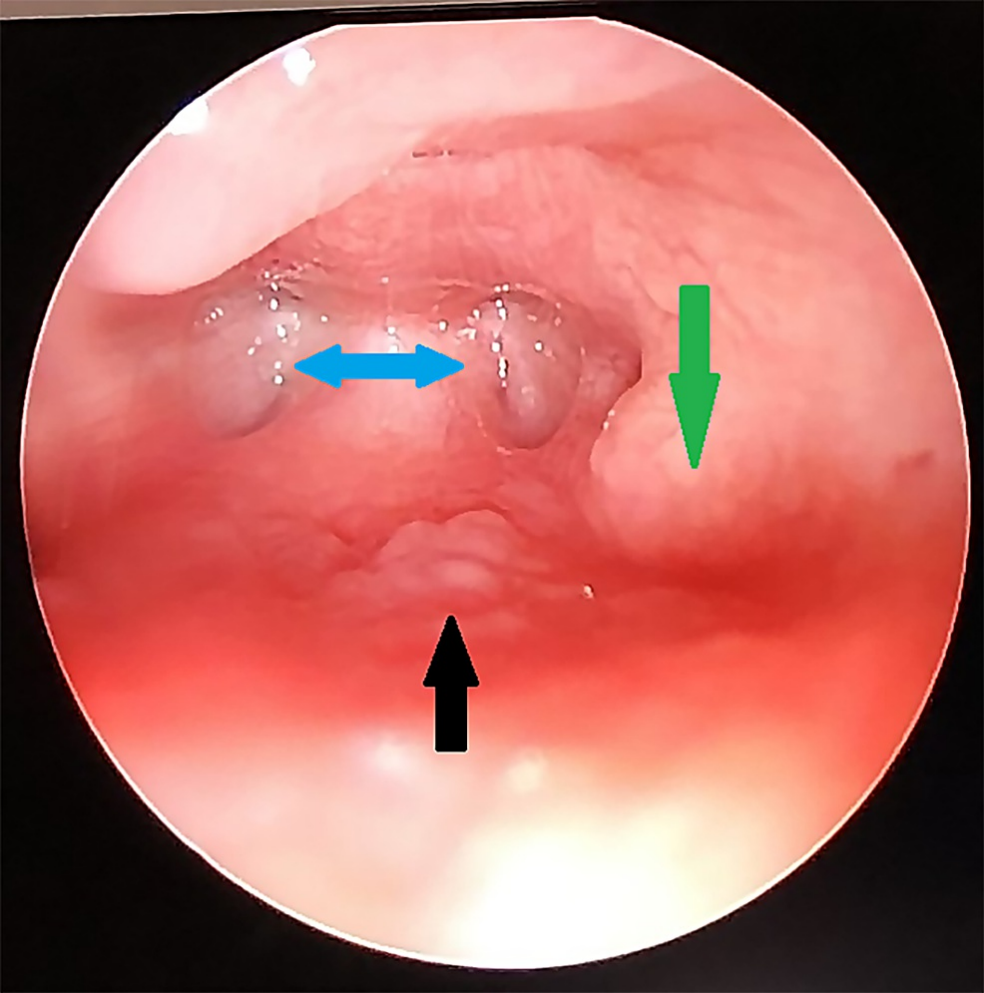
Nasopharynx viewed transorally using 120-degree Hopkins rod telescope. Intraoperative transpalatal view of the bilateral choanal atresia (blue arrows). This view was held by an assistant for visualization of the urethral sounds advancing through the atresia from the transnasal side. (Green arrow: left torus tubarius; black arrow: pharyngeal adenoid pad).

The patient was taken to the operating room for transnasal endoscopic repair. Utilizing two operating room screens, the choanae were viewed from both the nasopharyngeal and nasal sides. The patient was placed into suspension using a Dingman retractor. The primary surgeon utilized a transnasal endoscopic approach while a 120-degree Hopkins rod telescope was inserted transorally to view the bilateral stenosis from the nasopharyngeal side (Figure 2). The membranous atresia was reduced with a transnasal approach utilizing laryngeal suction cautery since the traditional suction cautery device was too large for the neonate’s nares. Next, serial urethral sound dilation was performed by carefully inserting serially larger urethral sounds through the bilateral bony atresia. Due to the severe stenosis level requiring a significant dilation, choanal stents were then fashioned from an endotracheal tube and left in place. These were removed in the operating room four weeks later. The patient was ultimately diagnosed with CHARGE syndrome, and she continues to be followed by the medical genetics and pediatric chronic care teams. Two years later, the patient’s mother continues to report excellent nasal breathing without any apparent concern for restenosis.

Case 2

**Figure 3:**
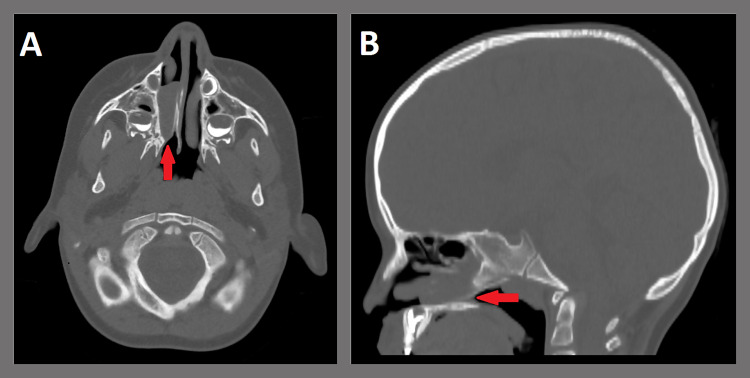
CT landmark sinus protocol without contrast (A: axial plane; B: sagittal plane). Note: Combined membranous and bony atresia of the right choana with a maximum diameter of 6 mm (red arrow). Mucinous secretions were seen throughout the right nasal cavity.

A healthy three-year-old female presented to the otolaryngology clinic with a lifelong history of right-sided nasal drainage unrelieved by intranasal steroids, oral antihistamines, or intranasal antihistamines. She denied any history of purulent discharge suggestive of sinusitis. Her mother denied any known history of coloboma, cardiac or genitourinary concerns, or hearing or speech concerns. Her BMI and growth history were appropriate for her age. Flexible nasoendoscopy revealed right-sided copious secretions and choanal atresia. CT landmark sinus protocol without contrast revealed mixed membranous and bony atresia of the right choana with a maximum bony diameter of 6 mm (Figure 3). The patient was taken to the operating room for successful endoscopic repair. The atresia repair was performed similarly to Case 1; however, unilateral choanal stenting was not performed. She was seen at a three-month follow-up with the resolution of rhinorrhea and no signs of restenosis. One year later, she remains free of symptoms.

## Discussion

Congenital choanal atresia is a well-described developmental anomaly characterized by an anatomical blockage of the posterior nasal airway with an estimated incidence between 1:5000 and 1:8000 births [1,2]. Unilateral atresia makes up about two-thirds of cases, with one-third being bilateral [2]. Similarly, one-third of cases are characterized by bony atresia, with the other two-thirds being mixed membranous and bony. Diagnosis is confirmed via both nasal endoscopy and computed tomography of the paranasal sinuses. 

Treatment of choanal atresia is exclusively surgical, as medical management is generally reserved for temporary relief of symptoms [2]. A current lack of randomized controlled trials leaves surgeons without direct evidence to help guide clinical decision-making and surgical approaches. One retrospective review by Brihaye P et al. of 36 patients showed favorable results with a one-stage surgical repair with local flap rearrangement [11]. Most authors have shown endoscopic transnasal techniques to be the most widely utilized surgical approaches, but the transpalatal approach can also be used with high success rates [1]. With either approach, delayed surgical repair is generally not recommended, especially in cases of bilateral atresia. A systematic review by Murray S et al. showed a significant increase in treatment failure rates with delayed repair versus immediate repair [12]. Other approaches have been described, including a transseptal approach involving the removal of portions of the vomer and medial pterygoid processes and the atretic plate [13]. In addition, authors have described supplemental treatments such as the application of mitomycin C concurrently with surgical repair [4]. However, there remains a lack of consensus or guidelines to guide surgeons on treatment approaches and adjunctive measures.

A total of 16 articles met the criteria for literature review (Table 1). Since the 2012 Cochrane Review, the most used approach to treat unilateral and bilateral choanal atresia remains endonasal endoscopic repair. A review and meta-analysis by El-Begermy MM et al. evaluated the transpalatal repair to the transnasal endoscopic approach, and there was no significant difference in restenosis rates noted [4]. According to Velegrakis S et al. and Attya H et al., factors affecting the restenosis rates in patients included bilateral choanal atresia and comorbidities like CHARGE syndrome [3,5]. Major complications of the procedure were described by case reports from D'Ascanio L and Rebuffini E and Bertossi D et al. that included a case of permanent unilateral blindness and traumatic optic neuropathy, respectively [14,15].

**Table 1:** Literature review and summary. PubMed, Google Scholar, and Scopus databases were queried for articles with “choanal atresia” and “surgery” in the title with a publication date between 2012 and 2021. Search results revealed 16 articles, summarized in the table. CA: Choanal atresia.

Author	Year	Article Type	Summary
Kuroda Y et al. [6]	2012	Case Report	64-year-old patient with acquired CA underwent transnasal endoscopic surgery, treated with mucosal flap, and stented for five days following the procedure. No stenosis evident following two years postoperatively.
Velegrakis S et al. [3]	2013	Retrospective Cohort	50 patients with CA were treated endonasally; recurrence rate was 57% in bilateral CA and 25% in unilateral CA. Higher rate of restenosis noted in those with bony atretic plates, CHARGE association, and bilateral CA. Stents had no effect on the recurrence rate.
Kinis V et al. [16]	2014	Retrospective Cohort	33 patients with CA, separated by diagnosis period, treated by transnasal endoscopic surgery and stents. Restenosis rate of 53.8% in group I (neonate) and 23.1% in group II (6 months after birth), not statistically significant. Overall success rate of surgery was 61.5%.
D'Ascanio L and Rebuffini E [14]	2014	Case Report	24-year-old presented with permanent, unilateral blindness following transnasal endoscopic surgery and stent of right unilateral CA. Stent was removed post-surgery, and three months later, the right nasal fossa had total fibrous obliteration.
Strychowsky JE et al. [10]	2015	Meta-Analysis	15 studies met inclusion criteria and found no difference in restenosis rates with or without the use of stents following endonasal repair. Stenting may be associated with a higher rate of complications. I can make no definitive statements regarding adjunctive mitomycin C due to limited data.
El-Begermy MM et al. [4]	2016	Meta-Analysis	No statistical significance of restenosis rate between transnasal endoscopic (35.7%) and transpalatal approach (28.2%). Mitomycin C and nasal stenting did not present a statistically significant difference in restenosis.
Holtmann L et al. [17]	2018	Retrospective Cohort	11 patients (7 unilateral CA and 4 bilateral CA) underwent transnasal endoscopic surgery. 27% restenosis in unilateral CA.
Bertossi D et al. [15]	2018	Case Report	Iatrogenic traumatic optic neuropathy following surgery for CA.
Murray S et al. [12]	2019	Systematic Review	Patients with bilateral CA experienced a significantly higher rate of treatment failure with delayed surgery (42.6%) compared to immediate surgery (24.8%), with no difference in mortality rate or respiratory function. Treatment outcomes were not affected based on surgery intervention timing in patients with unilateral CA.
Zeng L et al. [18]	2020	Retrospective Cohort	19 patients with a history of radiotherapy for nasopharyngeal carcinoma; 3 cases of unilateral CA and 16 cases of bilateral CA who underwent transnasal endoscopic surgery with nasoseptal mucoperiosteal flap. No failure was noted one-year post-procedure. Three patients with narrowing (<50%) of the choana.
Sung JY et al. [7]	2020	Case Report	19-year-old with Tessier number 3 cleft diagnosed with bilateral CA in adulthood. Treated with endoscopic sinus surgery using image-guided navigation system for opening and widening left CA, stent kept in place for eight weeks, and no restenosis evident after 26 months.
Rahman S et al. [8]	2020	Case Report	Two cases of babies with bilateral CA treated by endonasal endoscopy surgery with stents placed and removed after eight weeks. After 6 and 7 months of age, patients were reported to feed and gain weight normally.
Wilcox LJ et al. [9]	2020	Case Series	Five patients at a single institution underwent choanal atresia repair, and six drug-eluting stents were used among these patients. No cases of restenosis or stent-related complications were reported with a mean follow-up period of four months.
Attya H et al. [5]	2021	Case Series	42 patients with bilateral CA and unilateral CA, treated with transnasal approach, were evaluated over a 20-year period. Patients with bilateral CA, GERD, and comorbidities (e.g., syndromes) had a higher restenosis rate. Stents and mitomycin C did not decrease the restenosis rate.
Baldovin M et al. [19]	2021	Retrospective Cohort	39 children with bilateral CA who underwent endonasal endoscopic CA repair: restenosis rate was 31.3% in CHARGE population and 47.8% in non-syndromic CA cohort. A significantly increased number of dilations needed in stented patients of the non-syndromic cohort. Higher rate of restenosis in stented patients of both groups, though not statistically significant.
Siu AY et al. [20]	2021	Case Report	Presentation of an endonasal, endoscopic and stentless approach. Construct fold-over circumferential flaps in infants and cross-over flaps in older children. Use a mixture of 1% mometasone and 1% gentamycin ointment on the wound.

Although the endonasal endoscopic approach remains the most widely used, there remain differences in the surgical technique and management of choanal atresia regarding the use of mucosal flaps, stents, and mitomycin C. Siu AY et al. [20], Kuroda Y et al. [6], and Zeng L et al. [18] describe the use of mucosal flaps in their transnasal endoscopic surgery, and Kuroda Y et al. and Zeng L et al. report no restenosis occurrences. The effect of stents on restenosis rates is inconsistent. Velegrakis S et al., El-Begermy MM et al., and Attya H et al. showed that stenting did not affect restenosis rates [3-5]. However, Baldovin M et al. reported a higher rate of restenosis in stented patients with bilateral choanal atresia in the CHARGE syndrome and non-syndromic choanal atresia groups. Stented patients with non-syndromic choanal atresia required a significantly higher rate of dilations [19]. Despite the variation in restenosis rates, case reports by Kuroda Y et al., Sung JY et al., and Rahman S et al. describe the use of stents to successfully treat their patients [6-8]. Stenting also comes with risks and limitations. Using stents can increase the severity of postoperative management and contribute to stent-related complications, such as discomfort, infection, ulceration, scarring, and granulation tissue formation, which may require additional repair [1]. Following surgery, mitomycin C has been used to prevent restenosis, but in El-Begermy MM et al. and Attya H et al., mitomycin C did not affect the restenosis rate [4,5]. Use of mitomycin C has also been shown to decrease the amount of granulation tissue formation and the requirement for revision of surgical procedures. However, there is a lack of evidence on the long-term effects of using this treatment for benign pediatric conditions. Available evidence does not support the adjunctive use of mitomycin C [1]. The manuscript by Siu AY et al. reported the use of a mixture of 1% mometasone and 1% gentamycin ointment on the wound bed instead of mitomycin C [20].

This research manuscript is subject to limitations. The main limitation of this study is the availability and accessibility of published papers that match the specified search criteria developed for the literature review. Using the search methodology outlined in the “Materials and methods” section, 16 articles met the criteria, and no papers were excluded. This criterion was constructed to limit the scope of our study to include articles that discussed the surgical management of choanal atresia, but it could have overlooked other papers that may have been captured by different search parameters. An additional limitation of this study includes both its retrospective nature and the largely retrospective methodology of the studies included in the analysis. This limits the strength of this literature review and any conclusions inferred from the underlying data.

Overall, there was not enough evidence to definitively change previously published treatment recommendations. However, since the Cochrane Review, there has been more evidence suggesting a growing shift toward endoscopic approaches to surgery. Stenting continues to be a controversial issue, and there is a promise for adjunctive therapies like mitomycin C, which needs more evidence to support its routine use. This is complicated given the relatively low incidence of choanal atresia and difficulty in performing prospective studies.

## Conclusions

Choanal atresia is a rare congenital airway malformation that presents a unique surgical challenge for pediatric otolaryngologists. Definitive surgical management can be achieved with either endoscopic transnasal or transpalatal approaches. Although advances in surgical approaches and increased reporting of long-term outcomes are apparent since the 2012 Cochrane review, classically described transnasal or transpalatal surgical approaches remain the standard of care. In the cases presented herein, transnasal surgery with stenting was successful, but repair should always be performed using the approach most comfortable and familiar to the surgeon.
